# The essential role of hydrogen gas recycling by gut microbes in reducing deuterium load in host mitochondria: is trimethylamine oxide a deuterium sensor?

**DOI:** 10.1007/s11306-026-02443-3

**Published:** 2026-04-29

**Authors:** Stephanie Seneff, László G. Boros

**Affiliations:** 1 Computer Science and Artificial Intelligence Laboratory, Massachusetts Institute of Technology, Cambridge, MA 02139 USA; 2 The Deutenomics Science Institute, Los Angeles, CA USA

**Keywords:** Gut microbiome, Deuterium, Trimethylamine N-oxide, Atherosclerosis, S-Adenosyl methionine, Inflammation, 1C Metabolism

## Abstract

**Background:**

The human gut microbiome plays many essential roles, but an often-overlooked role is to maintain an abundant supply of deuterium depleted (deupleted) nutrients to fuel the host mitochondria. Excess deuterium (heavy hydrogen) damages mitochondrial ATP synthase nanomotors, leading to a decrease in matrix water production with increased reactive oxygen species (ROS) and inefficient ATP production. A microbial metabolite, trimethylamine N-oxide (TMAO) is a powerful signaling molecule whose plasma levels are high in association with many chronic diseases, including diabetes, fatty liver disease, and atherosclerosis, as well as cancer and dementia. Thus, TMAO is an important gut-host signaling molecule that serves as a marker for an imbalanced microbiome that is unable to fully metabolize trimethylamine (TMA), an important step in maintaining a deupleted nutrient supply.

**Aim of review:**

In this paper, we present a hypothesis that TMAO is a marker for deuterium overload in the methylation pathway, in addition to its role as an indicator of a disrupted gut microbiome. The original study that brought attention to TMAO involved feeding mice synthetic choline with fully deuterated methyl groups. Fully deuterated TMAO was subsequently detected in the plasma. By contrast, a diet rich in eggs, a natural source of choline (a precursor to TMAO), does not raise TMAO levels. Many of the pathologies that are linked to elevated TMAO can also be viewed as strategies to promote the supply of deupleted water to the mitochondria, systemically.

**Key scientific concepts:**

The mantra that “food is medicine” is well supported by the powerful role that gut dysbiosis plays in influencing human health and disease.

## Introduction

In the past two decades, researchers have become increasingly aware of the essential roles that the gut microbiome plays in maintaining the health of the host, in part by supplying many essential vitamins, amino acids, and other metabolites, and by metabolizing dietary fiber to produce short chain fatty acids (SCFAs) (Kumar et al., 2025). They also release signaling molecules that communicate with the brain through the gut-brain axis (Kim, 2024). Dysbiosis is defined as an imbalance in the gut microbiome, favoring pathogens over beneficial bacteria, which leads to an infiltration of immune cells and inflammatory bowel disease (IBD) (Zhang et al., 2025).

An important aspect of the metabolic processes in the gut and beyond is the methylation cycle, which originates in the production of 5,10-methylenetetrahydrofolate (CH_2_-THF) from THF and formaldehyde by bacteria, a key step in microbial one-carbon metabolism (Pietzke et al., 2020). Following the reduction of CH_2_-THF to methyltetrahydrofolate (CH_3_-THF) by CH_3_-THF reductase, the methyl group is transferred to the sulfur atom in homocysteine to produce methionine, which is then converted to S-adenosylmethionine (SAMe), considered to be the universal methyl donor (Matthews et al., 1998). SAMe covalently binds methyl groups to many molecular targets, including arginine and lysine residues in proteins (especially in histones), as well as cytosine and adenine bases in DNA and RNA (Menezo et al., 2020). The liver enzyme phosphatidylethanolamine methyl transferase (PEMT) transfers three methyl groups from SAMe to phosphatidylethanolamine to produce phosphatidylcholine (Li et al., 2023). SAMe also contributes methyl groups in neurotransmitter production.

DNA and histone methylation regulate epigenetic modifications and imprinting. Phosphatidylcholine is a precursor to acetylcholine, an excitatory neurotransmitter in the brain. Trimethylated lysine molecules, recovered during protein metabolism, are precursors to L-carnitine, which retains the trimethylamine moiety (Shekhawat et al., 2013). L-carnitine facilitates the transport of fatty acids into the mitochondria to be oxidized for fuel production. Dietary phosphatidylcholine and dietary L-carnitine, in addition to the endogenous sources, are important nutrients that provide methyl groups to the methylation cycle. Choline and L-carnitine, as well as a close relative, betaine (trimethylglycine), are all precursors to trimethylamine (TMA), a small methylated amine produced through microbial enzymatic action (Tang & Hazen, 2014; Heinrich-Sanchez and Vital, 2025). Importantly, obligate anaerobic hydrogen-dependent archaea called methylotrophs in the gut can reduce the three methyl groups in TMA to methane gas, using molecular hydrogen as a reducing agent, or to formaldehyde, catalyzed by the enzyme TMA dehydrogenase (TMADH) (Jang et al., 1999; Feldewert et al., 2020). This formaldehyde can then launch a new cycle in the methylation pathway, through binding to THF. Ammonium (NH_4_^+^) is released as a side product.

Another significant pathway involving the gut microbiome also has a cyclic component, whereby gut microbes consume dietary nutrients and sulfomucins produced by the host cells, and convert them to SCFAs: acetate, propionate and butyrate. Butyrate is a preferred fuel for the colonocytes lining the gut, providing up to 70% of their energy needs (Recharla et al., 2023). Dietary fiber is fermented by anaerobic bacteria in the gut, particularly species within the Bacteroides, Bifidobacterium and Lactobacillus genera, yielding molecular hydrogen and carbon dioxide (CO_2_) (Cronin et al., 2021). The hydrogen gas is then used as a reducing agent to convert CO_2_ into SCFAs (Welsh et al., 2025). Goblet cells lining the colon produce highly glycosylated and sulfated proteins called sulfomucins, which are thought to be the main contributor to the elasticity and viscosity of mucus (Wagner et al., 2018). The mucus layer of patients with ulcerative colitis is thinner than normal, especially in areas of inflammation, along with a reduction in the number of goblet cells and a loss of integrity of the mucus layer. Breaching of the barrier by pathogens and their endotoxins, such as lipopolysaccharides (LPS) further promotes inflammation in a positive feedback loop. The mucus layer is often severely degraded or missing in areas of acute inflammation (Pullan et al., 1994).

*Akkermansia mucinophila* is a gut colonizer whose sole source of nutrients is the sulfomucins lining the gut barrier. In a symbiotic relationship with the host, *A. mucinophila* break down sulfomucins, and, with the help of other microbes such as Firmicutes, convert them back to SCFAs, especially butyrate (Effendi et al., 2022). This process contributes to a healthy gut barrier and overall metabolic and immune function (Mo et al., 2024). Furthermore, *Akkermansia* produces an 84 kDa protein called P9 that induces the secretion of glucagon-like peptide 1 (GLP-1) by L-cells in the colon (Yoon et al., 2021). GLP-1 is a natural gut hormone that helps regulate blood sugar, digestion, and appetite. Butyrate also induces GLP-1 upregulation (Yadav et al., 2013). The pharmaceutical industry has been very successful in promoting GLP-1 analogs such as Wegovy and Ozempic to treat diabetes and promote weight loss (Watanabe et al., 2024).

## Evidence that deuterium disrupts mitochondrial function

Deuterium (^2^H) is a heavy isotope of protium (^1^H; hydrogen), and it is pervasive in nature, found in seawater at a concentration of 155 parts per million (ppm) relative to protium. Deuterium is highly damaging to the F_1_F_0_-ATP synthase (ATPase) nanomotors in the mitochondria that produce ATP, the primary fuel source of the cell (Olgun, 2007). The exchange of a proton with an ionized deuteron in bio-molecules is called deuteronation. This is a common stochastic process in physiological deupleted nutritional states that aligns the two ATP synthase c subunits for optimal performance with broad implications for health (Lanjanian et al., 2026). Deuterium loading suppresses the activity of many fundamental biologically important hydrolytic enzymes that depend on proton tunneling. Notably, it is likely that deuterium increases the frequency of unrepaired nuclear DNA mutations, by suppressing the activity of deuterium-sensitive repair enzymes (Yasuda et al., 2024).

The inherent collective proton tunneling (ICPT) process, which uses membrane-bound ATPase nanomotors in living organisms, is nature’s ultimate tool for discriminating hydrogen isotopes. This is because a deuteron (^2^H) cannot replace a proton (^1^H) in its tunnel protein during enzymatic transmembrane transport (Kotyk et al., 1990) due to its doubled mass and twice larger atomic nuclear size. The result is large compartmental, inter- and intramolecular deuterium disequilibrium in ^2^H/^1^H ratios (Boros et al., 2024) in all biomolecules, which readily distinguishes respiration from aerobic fermentation (Maloney et al., 2024) with adaptive significance. In essence, deuterons are retained for DNA instability and biomass production in prokaryotes (Sobczyk et al., 2013), and for structural amino acids in the connective and supportive tissues of eukaryotic organisms (Gharibi et al., 2022).

Simultaneously, deuterons irreversibly clog single proton tunneling ATP synthase nanomotors in the mitochondria (Olgun, 2007), according to conformational subunit c alignments (Lanjanian et al., 2026). The result is the complete breakdown of inherent collective proton tunneling, which results from deuterium’s exceptional isotopic-substitution effect (Drechsel-Grau & Marx, 2014) in interfacial structured water, such as that found in mitochondria (Ford et al., 2004). This initiates many disease-causing molecular crowding mechanisms, which we review herein from the perspective of prokaryotic proton pumping and H_2_ gas formation in the organic molecular realm of mitochondrial proton-donating substrates. Understanding at the systems level how humans protect mitochondrial ICPT processes and ATP synthase is a fascinating journey reviewed herein.

We hypothesize that the process uses TMAO as the microbial stepping stone, employing deuterium discrimination to become an active player in forming the biological reaction coordinate via remote deupleted mitochondrial water formation in a healthy host (Zhang et al., 2020).

Table 1 shows energy dissipated as heat during protonated mitochondrial matrix water formation in absolute kJ/mol values by TMAO-driven metabolic hydrogen peroxide and water formation reactions in cells in comparison with that of ATP formation. Values are given as standard enthalpy of formation from H_2_, O_2_ and adenosine diphosphate (ADP). Values are calculated as ΔH^o^_reaction_ = ΣH^o^_products_ – ΣH^o^_reactants_, which reflect new (net) formation of product metabolites shown in Table 1 (Alberty 2000; Boros et al. 2024a). Considering the unit of Joule (J) heat energy, which is released when an electric current of one ampere passes through a resistance of one Ohm for one second, 1 mol (18gr) of deupleted water formation yields considerable heat measured in kJ units, coupled with 1.5 moles of ATP’s mechanical (rotational) energy invested in nanomotor functions. Deupleted microbial TMA is likely one of the most important precursors of L-carnitine through hydrogen recycling, and, subsequently, deupleted fatty acid signaling and transport through cell membranes and mitochondria, to produce highly energetic mitochondrial products (e.g., ATP) essential for healthy human life. For additional fitting of mitochondrial ATPase models please see Bennett & Onyango, 2021.

### Quantum tunneling and proton-coupled electron transport

Inherent collective proton tunneling is a theoretical quantum mechanical phenomenon proposing that a single proton spontaneously passes through a potential energy barrier, typically within a hydrogen bond, in a manner that can be functionally irreversible. Unlike classical particles that must surmount energy barriers, protons can “tunnel” through them due to their wave-like nature. Often, this single proton tunneling is part of a larger process where a proton and an electron are transferred simultaneously (or sequentially) as a single kinetic step, often in the presence of strong electric fields that stabilize the transferred state. This process is referred to as “proton-coupled electron transport” (PCET) (Klinman et al., 2013; Layfield & Hammes-Schiffer, 2014).

Enzymes that phenomenally speed up a reaction involving C-H bond cleavage by up to a factor of 10^15^ almost certainly exploit proton tunneling to achieve this feat (Korchagina et al., 2025). Mitochondria exploit PCET to build the proton gradient that powers the nanomotors to produce ATP in the electron transport chain (ETC). Impaired ETC activity in mitochondria in the brain is linked to neurodegenerative disease (Bennett & Onyango, 2021). Many enzymes, such as dehydrogenases and lipoxygenases, exploit proton tunneling to carry out their reactions. Deuterons are much less capable of tunneling, so this becomes a way to select for substrates containing protons rather than deuterons. The very large kinetic isotope effect (KIE) (81) for soybean lipoxygenase is an example of this phenomenon (Knapp, et al., 2022).

Complexes I, III, and IV act as redox-driven pumps, exploiting mitochondrial ICPT to transfer protons from the matrix into the intermembrane space. NADH-ubiquinone oxidoreductase (Complex I) couples the transfer of two electrons between NADH and ubiquinone to the translocation of four protons across the membrane (Sazanov, 2014). This process provides the driving force for ATP synthase, which harnesses the gradient to produce ATP, but it also assures that few, or no, deuterons arrive on the other (intermembrane) side of the membrane, protecting the ATPase nanomotors.

SAMe, the universal methyl donor, plays a crucial role in regulating oxidative phosphorylation (OXPHOS). It is primarily synthesized in the cytoplasm and imported into the mitochondria via the import protein SAMC. Homozygous mutations in *Slc25A26*, the gene that encodes SAMC, are embryonically lethal in mice (Rosenberger et al., 2021). These authors wrote: “SAMC is the only mitoSAM carrier and is required for OXPHOS and oxidative tricarboxylic acid (TCA) metabolism,” showing a strong dependency of mitochondrial health on  1C metabolism. We hypothesize that the importance of SAMe to mitochondrial health is directly linked to the plausible theory that SAMe’s methyl groups are normally highly deupleted.

### Deuterium disrupts ATPase nanomotors

Hydrogen/deuterium exchange experiments have demonstrated that deuterium accumulates in and destabilizes the γ-rotor shaft in F1-ATPase. Deuterium becomes trapped in the C-terminal helix, which leads to an unfolding of the helix and a stalling of the motor (Vahidi et al., 2016; Murcia Rios et al., 2018). This results in an inefficiency in ATP production and an increased release of reactive oxygen species (ROS) (Olgun, 2007).

### Deuterium suppresses DNA double-strand break repair processes

One of the most frequent types of damage to a pyrimidine base in DNA is the spontaneous deamination of cytosine to generate uracil. In mitochondria, UNG (Uracil-DNA Glycosylase) is responsible for the first step of Base Excision Repair (BER) for uracil. It recognizes uracil misincorporated into DNA through deamination of cytosine and cuts the glycosidic bond to remove it (YJ Kim et al., 2012).

The *UDG* gene encodes both the mitochondrial (UNG1) and the nuclear (UNG2) forms of human UDG (Akbari et al., 2008). Krokan et al. (2001) wrote in the abstract that “Ung-/- cells are deficient in removal of misincorporated dUMP and accumulate approximately 2000 uracil residues per cell” (Krokan et al., 2001).

UNG has large primary and secondary β-deuterium KIEs (Werner & Stivers, 2000). This suggests that excess deuterium loading in the mitochondria would significantly impair the ability to repair mitochondrial DNA damage, which is compounded with the increased release of ROS from deuterium-loaded ATPase nanomotors. These observations support the fact that cancer cells, which likely carry an increased deuterium burden (Seneff and Kyriakopoulos 2025a; Kyriakopoulos and Seneff 2026), have high rates of mutation in mitochondrial DNA (Brandon et al., 2006).

### DDW is an effective cancer treatment option

Evidence continues to build through many papers on deuterium depleted water (DDW) as a therapeutic option for cancer treatment, and it has been shown that this simple treatment prolongs lifespan in cancer patients (Kovács et al., 2022; Lu & Chen, 2024). It has been demonstrated experimentally that an elevated deuterium concentration in the medium (300 ppm) acts as an active driver of cancer progression in lung adenocarcinoma cells, causing a significant increase (up to 2.1-fold) in the expression of many oncogenic genes involved in tumor survival, proliferation, and metastasis (Csonka et al., 2025).

## Are microbially synthesized methyl groups and butyrate deuterium depleted?

A careful tracing of multiple metabolic processes taking place in a human cell reveals that they are plausibly designed to greatly restrict the number of deuterons that are in the mitochondrial water. This strategy helps to minimize exposure of the ATPase nanomotors to deuterons. In part, this feat is accomplished through enzymes such as flavoproteins that greatly favor protium over deuterium in their reaction, i.e., that have a high deuterium KIE (Sutcliffe et al.,^2002^). The physics usually involves configuring the enzyme to support proton tunneling, since deuterons are much less capable of such tunneling (Henkel et al., 2014).

Another way to support a reduced deuterium supply to the ATPase nanomotors is to select nutrients that are naturally low in deuterium to feed into the tricarboxylic acid (TCA) cycle. This is what makes the metabolites produced by the gut microbes via hydrogen recycling very significant. The molecular gas H_2_, produced through anaerobic fermentation via catalytic activity of a hydrogenase expressed by *Pseudomonas* sp, was shown to be 80% depleted in deuterium (Krichevsky et al., 1961). This likely also holds for hydrogenases expressed by coliform bacteria inhabiting the human gut (Vignais, 2008). It likely happens in part because ^2^H, being twice as heavy, prefers to stay behind in the aqueous phase. Furthermore, microbial [NiFe] hydrogenase has a high KIE for deuterium extraction, as high as 43 in an acidic environment (Greene et al., 2015). Methane and acetate are then produced by reducing CO_2_, using H_2_ as the reducing agent, by methanogens and acetogens, respectively.

In subsequent reactions, protons bound to carbon atoms in methane and acetate are transferred to NAD^+^ to yield NADH, often involving enzymes that have a high deuterium KIE. Finally, NADH dehydrogenase (Complex I) pumps protons from the matrix into the intermembrane space, while restoring NAD^+^, and maintaining low deuterium in the mitochondrial water (Seneff et al. 2025a).

In another pathway, methane is converted to formaldehyde, which carries two deuterium depleted (deupleted) protons that are incorporated into the methylene unit bound to THF in CH_2_-THF. CH_2_-THF reductase (MTHFR) adds a proton taken from deupleted NADPH to finally produce CH_3_-THF, which fuels methyl groups into the methylation cycle (Seneff and Kyriakopoulos 2025a). MTHFR is a flavoprotein which has a deuterium KIE of about 2.9 (Schmidt et al., 2000). Overall, methyl groups produced through these catalytic processes are expected to be extremely deupleted.

The enzyme expressed by anaerobic archaea that metabolize TMA, TMADH, is a flavoprotein with a high deuterium KIE (~ 8.6) due to vibrationally assisted hydrogen tunneling (Basran et al., 2001; Wanninayake et al., 2019). This means that the hydrogen recycling that takes place during its metabolism further scrubs deuterium from the methylation pathways, while the TMA that is left behind becomes enriched in deuterium. This unmetabolized TMA is converted to deuterium-enriched trimethylamine N-oxide (TMAO) in the liver and released into the circulation. Elevated TMAO levels in plasma are associated with increased risk to cardiovascular disease and a long list of other inflammatory diseases (Constantino-Jonapa et al., 2023), as we will detail in the coming sections.

## Natural and synthetic choline have different effects on TMAO levels: does deuterium play a role?

The original paper that first identified TMAO as a risk factor for heart disease, published in 2011, involved feeding mice phosphatidylcholine where all the protons in the methyl groups attached to the nitrogen atom were replaced with deuterium, so that the researchers could trace the products of the nutrient in the body (Wang et al., 2011). It turns out, then, that they accidentally conducted an experiment testing what happens when phosphatidylcholine is extremely enriched in deuterium. The D9-PC was formed by methylating naturally produced phosphatidylethanolamine in the chemistry lab using fully deuterated methyl groups. The title of their paper was: “Gut flora metabolism of phosphatidylcholine promotes cardiovascular disease.” Supplementation of mice with D9-PC resulted in D9-TMAO present in plasma. They also determined that supplementation of mice with deuterated choline, TMAO, or betaine resulted in the upregulation of multiple macrophage scavenger receptors linked to atherosclerosis. TMAO was not produced if the mice were pretreated with antibiotics or in experiments with germ-free mice, confirming that microbial enzymatic action was a necessary precondition.

A paper published by J. Wilcox et al. in 2021 on human subjects compared choline intake from natural dietary sources with supplemental choline bitartrate and found that the latter but not the former raised blood TMAO levels. Notably, these authors wrote in the conclusion of the abstract: “Despite high choline content in egg yolks, healthy participants consuming four eggs daily showed no significant increase in TMAO or platelet reactivity” (Wilcox et al., 2021). However, TMAO levels rose significantly following synthetic choline bitartrate supplementation. This occurred even though the subjects had normal kidney function, showing that elevated TMAO is not just a consequence of kidney disease.

The authors of this study suggested that it might be that phosphatidylcholine is processed differently than choline bitartrate by the gut microbes. However, deuterated but not natural PC does raise plasma TMAO levels. The authors of the original 2011 study had published a follow-on study on human subjects in 2013, in which they supplemented the subjects with D9-PC, essentially repeating the mouse study but with humans as the subjects. They confirmed that D9-TMAO levels were sharply elevated in the plasma and urine following supplementation. Furthermore, an elevated TMAO level predicted an increased risk of major cardiovascular events, after adjustment for traditional risk factors (*P* < 0.01) (Tang et al., 2013). This study shows that it may not be phosphatidylcholine vs. choline bitartrate that matters, but rather whether the choline is deuterium depleted or deuterium enriched. By contrast, a survey involving over 14,000 participants found that dietary choline *protects from* both heart disease and stroke (Zhou et al.,^2023^).

An important follow-on research topic would be to conduct controlled experiments comparing outcomes in human or rat studies where natural PC supplementation is directly compared with an equivalent amount of deuterated PC supplementation. Such studies have not yet been done, mainly because researchers are unaware that there might be a health issue with deuteration.

L-carnitine is also a precursor to TMAO, and a mouse study in which the mice were fed deuterated L-carnitine also showed a sharp increase in plasma TMAO following supplementation, further supporting the idea that deuteration is the primary factor promoting TMAO accumulation (Koeth et al., 2013).

## TMAO directs metabolism towards deupleting peroxisomal-mitochondrial crosstalk

**Fig. 1:**
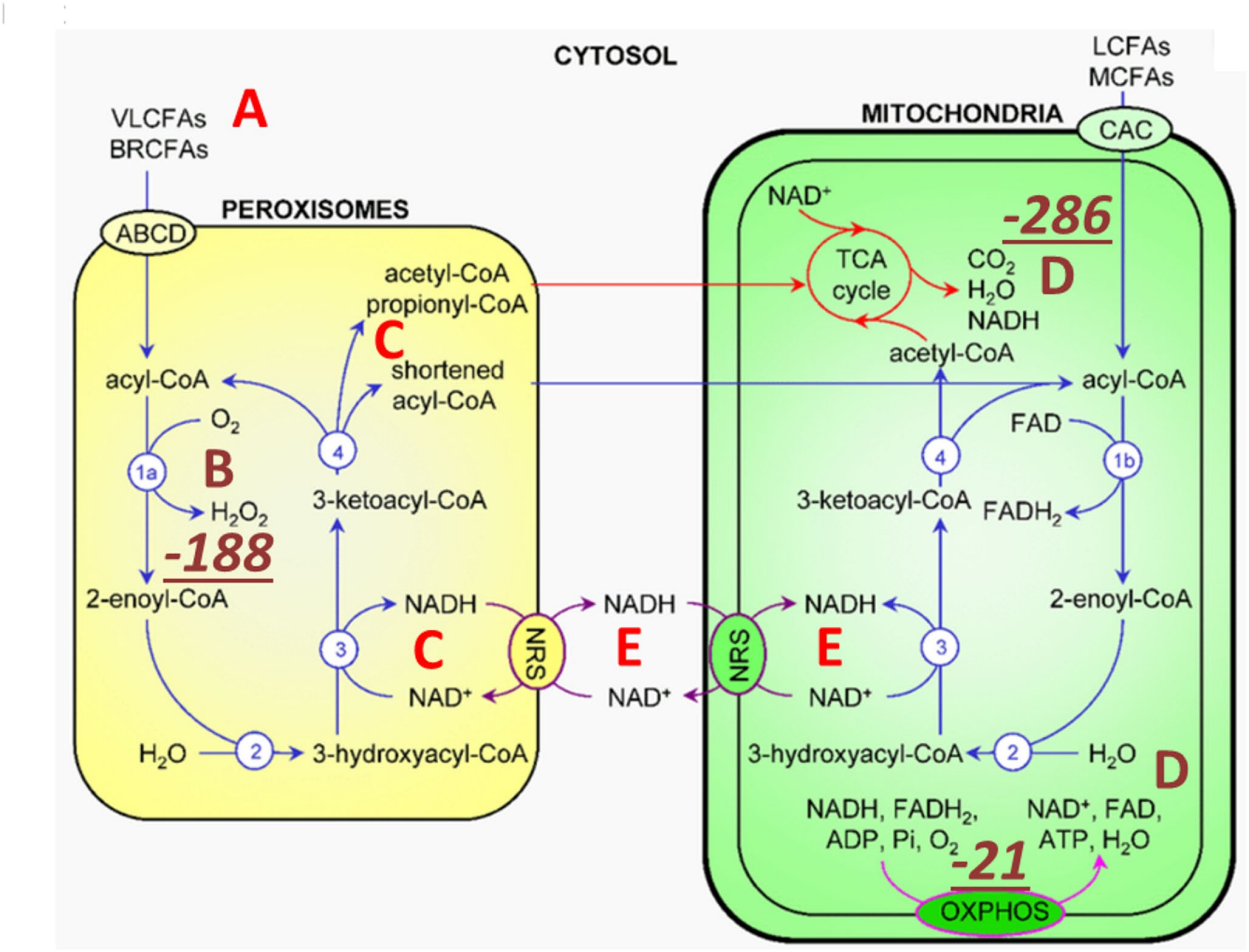
Metabolic cross talk and energy yield balance between peroxisomes and mitochondria. Peroxisomal metabolism triggered by TMAO turnover utilizes very long and branched chain fatty acids (**A**) as well as dissolved molecular oxygen (**B**) carried in plasma. Peroxisomes produce SCFAs via β-carbon oxidation, ketones, NADH (**C**) and hydrogen peroxide (**B**). H_2_O_2_ is rapidly converted to metabolic water by catalase (CAT) that also yields molecular oxygen for the mitochondrial matrix (**D**) as well as for other cellular compartments. Peroxisomes can also reduce NAD^+^ for proton delivery to mitochondria via membrane-based intracellular proton transporters. Energy yield (enthalpy) of exothermic metabolic reactions in kJ/mol values during peroxide, water and ATP formation are also shown (Alberty, 2000). ABCD: ATP-binding cassette transporters of subfamily D; NADH: Nicotinamide Adenine Dinucleotide; VLCFAs: Very Long Chain Fatty Acids; BRCFA: Branched Chain Fatty Acids; NRS: NAD(H) Redox Shuttles. Joule (J): the energy dissipated as heat when an electric current of one ampere passes through a resistance of one ohm for one second. (Image: Share and Cite Fransen et al. 2017; Boros et al. 2024a)

The synthesis of TMAO from TMA requires hydrogen peroxide (Pearson & Y. Yamamoto, 2001), according to the (CH_3_)_3_N + H_2_O_2_ → H_2_O + (CH_3_)_3_NO stoichiometry. The source of hydrogen peroxide is via peroxisomal fatty acid chain modifications that utilize molecular oxygen dissolved in plasma to produce SCFAs, ketones, NADH and hydrogen peroxide (H_2_O_2_). The resulting metabolic water of the reaction is deupleted, as fatty acids are inherently deupleted molecules in biology (Evans & S Beharie, 2024). Although peroxisomes do not produce ATP directly, they reduce NAD^+^ for proton delivery to mitochondria via membrane-based intracellular proton transporters. Peroxisomes operate with molecular oxygen dissolved in blood at concentrations of 3 ml/L or less (Lambertsen et al., 1953). As tissues at rest draw 50 to 60 ml of oxygen per liter of blood, assuming normal perfusion (Richalet et al., 1999), it is crucial to retain, supplement, and recycle water and oxygen via peroxisomal and mitochondrial metabolic crosstalk during fatty acid remodeling, with the help of TMAO turnover, as shown in Fig. 1.

Although the partial contribution of TMAO synthesis to intermediary metabolism is yet to be determined to efficiently deplete deuterium, it is certain that none of the above works well in the deuterium preserving glucogenic metabolic state. Intermembrane derived protons, due to the high inner membrane gradient, also power ATP synthase nanomotors, which are sensitive to deuterium (Olgun, 2007), diminishing their function. There is a strict dependence of peroxisomes on long chain saturated fatty acid substrates with particularly lower deuterium-related chemical mass (Répás et al., 2025), i.e., the isotopic composition of a molecule, also called molar isotope enrichment.

**Table 1:** Enthalpy (energy yield) of protonated mitochondrial matrix water in absolute kJ mol^− 1^ values during TMAO-driven metabolic hydrogen peroxide and water formation reactions in cells (Alberty 2000; Boros et al. 2024a)

Chemical formula	State of matter	H^o^: enthalpy**
H_2_O (mitochondrial water)	liquid	|285.83|
H_2_O_2_ (hydrogen peroxide in peroxisome)	liquid	|187.78|
C₁₀H₁₆N₅O₁₃P₃ adenosine triphosphate ATP	solid in solution	|20.5|*

*ATP synthase’s subunit c alignment and rotation efficiencies are strict deuterium discrimination dependent processes (Lanjanian et al., 2026) via prokaryote-derived TMAO deutenome (Boros et al. 2024b; Zhang et al. 2020).

The oxidation of very long chain saturated fatty acid β carbons, purportedly of animal source, with the help of molecular oxygen, yields the most deupleted H_2_O_2_ by weight (Fig. 1. B). CAT (EC 1.11.1.6), one of the fastest enzymes in biology with that of isomerases, rapidly and irreversibly produces water from H_2_O_2_, while recycling oxygen. Metabolic hydrogen peroxide of fatty acid breakdown with low deuterium consequently provides ATP synthase nanomotor-sparing protons for energy production (Seneff and Kyriakopoulos 2025b). High TMAO with hydrogen peroxide turnover can easily depend on CAT-mediated oxygen recycling. This is because the catalytic process of CAT obeys Michaelis–Menten kinetics, with a Michaelis constant equal to that for the catalytic reaction, i.e., the catalytic reaction of CAT, in the limit of zero reaction time (Boros et al. 2024a). It has also been confirmed that catalytic activity is substantially independent of pH in the range 4.7–10.5 that makes TMAO-induced CAT a safeguard to operate in the generally low pH medium, characteristic of metabolic ketosis (Jones & Suggett, 1968).

## Is TMAO an indicator of deuterium overload in the mitochondria?

In previous work, we have argued that molecular hydrogen recycling by gut microbes is an essential process for the generation of deupleted nutrients for the host (Seneff et al. 2025a; Seneff and Kyriakopoulos 2025a, c). TMA that remains unmetabolized by gut microbes becomes enriched in deuterium due to the large primary deuterium KIE (~ 8.6) of trimethylamine dehydrogenase, the microbial enzyme that metabolizes it (Wanninayake et al., 2019). Once it is passed on to the liver and oxidized there, it enters the blood stream as TMAO, which appears to be a very powerful systemic signaling molecule associated with many chronic diseases, including cardiovascular disease, diabetes, and fatty liver disease, among others.

There is little controversy around the idea that TMAO is not just a marker but a direct cause of disease through multiple toxic effects. Liu et al. (2025) wrote: “TMAO not only induces endothelial dysfunction but also acts on various cell types, such as endothelial cells, epithelial cells, vascular smooth muscle cells, nerve cells, and pancreatic cells, triggering multiple cell death mechanisms, including necrosis and programmed cell death, thereby influencing host health.” And, in the conclusion, they wrote: “TMAO may drive the cross-talk between inflammatory response and oxidative stress by mediating cell ‘death’ (including apoptosis, pyroptosis, autophagy, and ferroptosis, etc.) and then induce pathological processes such as foam cell activation, massive secretion of cytokines and adhesion molecules, overaccumulation of ROS, enhanced platelet reactivity and abnormal vascular tone regulation. It is deeply involved in the pathogenesis and progression of diseases across the ‘gut-organ’ axis: pulmonary diseases, including cardiovascular diseases (such as atherosclerosis, heart failure, and hypertension), renal diseases, neurodegenerative disorders, and metabolic diseases, including diabetes.”

According to a review article published in 2018, TMAO enhances the risk of systemic inflammation, diabetes, obesity, and atherosclerosis. It triggers thrombosis and is toxic to the kidneys. Elevated levels of TMAO are associated with heart failure and colorectal cancer (Subramaniam & Fletcher, 2018). We hypothesize that TMAO serves as a signal to the host of systemic mitochondrial deuterium overload due to an imbalanced gut microbiome.

Elevated levels of serum TMAO have been reported to be tightly related to atherosclerosis, due to its ability to induce inflammation, platelet activation, monocyte recruitment to the artery wall, and cholesterol accumulation in arteries. Studies show that high TMAO is associated with a greater risk of cardiovascular events, increased atherosclerotic plaque burden, thrombus generation, and a poorer prognosis for patients with cardiovascular disease (Zhu et al., 2016; Amaritei et al., 2025). This suggests to us that the accumulation of lipid-laden foam cells in the artery wall may be a protective mechanism to deplete deuterium in the circulation, a topic we will return to later.

### TMAO inhibits S-adenosylhomocysteine hydrolase

Whenever S-adenosylmethionine (AdoMet) donates a methyl group to a methyl acceptor via a methyltransferase, S-adenosylhomocysteine (AdoHcy) is produced as a by-product. The enzyme AdoHcy hydrolase then splits AdoHcy into adenosine and homocysteine, and it is the only enzyme that performs this activity. Methionine synthase then restores the methyl group, and methionine adenosyltransferase regenerates S-adenosylmethionine, available to methylate another acceptor. When AdoHcy hydrolase is suppressed, AdoHcy accumulates, and it acts as a powerful feedback inhibitor of methyltransferase. This essentially reduces methylation capacity, causing hypomethylation of various targets, such as nucleic acids, proteins, and lipids, associated with disease (Tehlivets et al., 2013).

TMAO inhibits AdoHcy hydrolase, leading to disrupted one-carbon metabolism, altered histone modifications, and other epigenetic effects (Han et al., 2025). The proteomes and chromatin states in the cortex and hippocampus were remarkably remodeled under exposure to high TMAO, which is expected to impact cognition and neurological health (Han et al., 2025). It is perhaps plausible that methylation pathways should be suppressed if the methyl groups are deuterium enriched, because methyl groups are ultimately expected to supply deupleted protons to mitochondrial water (Seneff et al. 2025a). The methyl groups will be deuterium rich if the recycling by anaerobic archaea via the production of H_2_ is impaired.

### TMAO induces reactive oxygen species

There is a growing body of literature that characterizes mechanistically how TMAO acts to induce production of ROS, leading to the upregulation of cytokines and chemokines, which then act to cause tissue damage in affected organs and to promote the progression of atherosclerosis.

Hyperlipidemic acute pancreatitis is an acute abdominal disease that is increasing in prevalence worldwide (Zhou et al., 2024). In a study involving a mouse pancreatic acinar cell cancer line exposed to TMAO, it was found that TMAO decreased cell viability and facilitated apoptosis in a dose-dependent manner. Levels of H_2_O_2_, ROS, nitric oxide, and superoxide dismutase (SOD) were all increased with exposure (Yang & X Zhang, 2021).

Elevated ROS levels in the vascular wall is a key pathological feature in atherosclerosis. NADPH oxidase (NOX) is a major ROS-generating enzyme class. NOX4 is abundantly expressed in both endothelial cells and vascular smooth muscle cells (VSMCs). In a mouse model, it was demonstrated that a Western diet combined with disturbed blood flow induced upregulation of NOX4 in mouse VSMCs, and this led to lipid accumulation in the vessel wall (Tong et al., 2016).

Protein arginine methyltransferase 5 (PRMT5) is a highly conserved enzyme that regulates gene expression through methylation of arginine residues in critical proteins. Specifically, PRMT5 symmetrically dimethylates the p65 subunit of NF-κB on arginine 30, which results in NF-κB activation and upregulated expression of many cytokine and chemokine genes (Wei et al., 2013). Vascular Cell Adhesion Molecule 1 (VCAM-1) is a crucial cell surface protein on activated endothelial cells that acts like a docking station, facilitating the binding of monocytes and lymphocytes to blood vessel walls and the subsequent migration from the blood stream into tissues during inflammation (Sans et al., 1999).

A seminal paper published in 2022 clearly demonstrated that TMAO exposure to VSMCs markedly induced expression of NOX4 and production of ROS, which upregulated PRMT5 and induced subsequent VCAM-1 expression (Liu et al., 2022). This constitutes a critical step in the seeding and progression of atherosclerotic plaque.

### TMAO suppresses autophagy via PI3K/Akt/mTOR activation

Autophagy serves a pro-survival function in the endothelium. Endothelial autophagic flux limits atherosclerotic plaque formation by protecting from endothelial apoptosis, senescence and inflammation (Kheloufi et al., 2018). Activation of the phosphoinositide-3 kinase (PI3K)-Akt-mammalian target of rapamycin (mTOR) signaling pathway can promote necrotic cell death by suppressing autophagy (Wu et al., 2009). An in vitro study which involved growing VSMCs in culture and exposing them to oxidized low-density lipoprotein (Ox-LDL) with or without TMAO exposure, while comparing them to unexposed cells, revealed that TMAO suppressed autophagy by activating the PI3K/Akt/mTOR pathway, increasing the levels of markers of atherosclerosis. Pretreatment with a recognized PI3K inhibitor reversed the effect (Shi et al., 2023). TMAO exposure also decreased the expression of Beclin 1, a key protein involved in autophagy initiation (Kang et al., 2011).

**Fig. 2:**
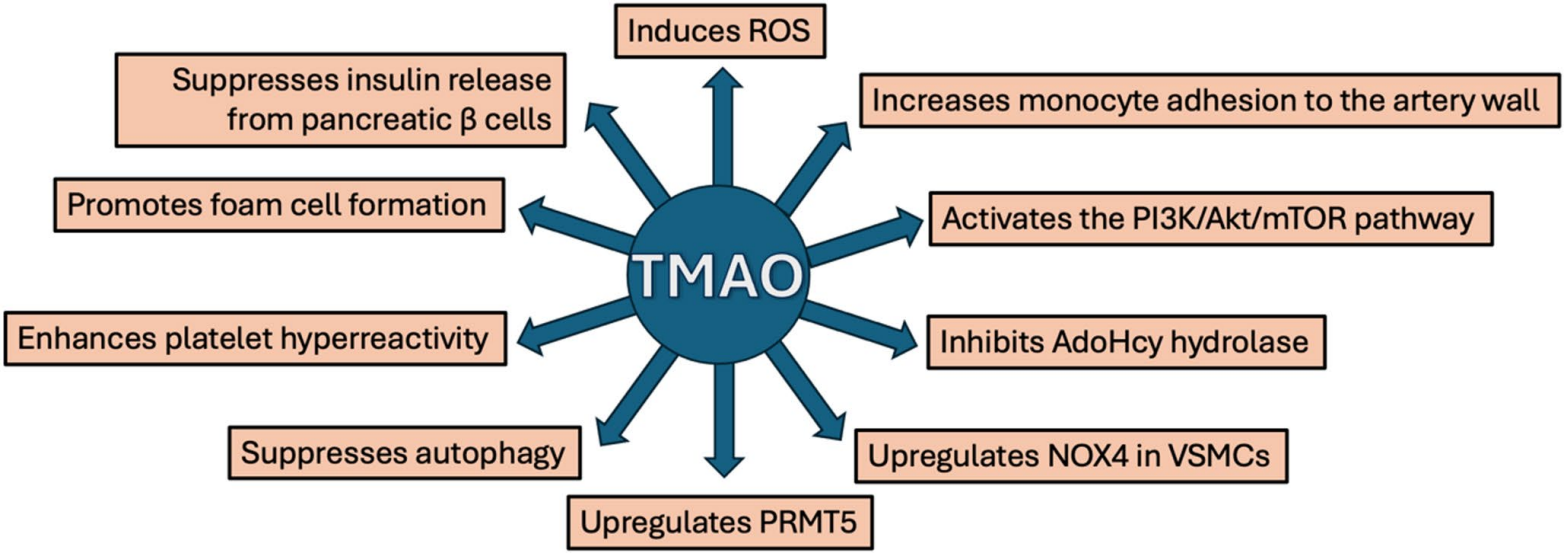
Specific mechanisms by which TMAO influences cellular metabolism to cause disease. ROS: Reactive Oxygen Species; P13K/Akt/mTOR: Phosphatidylinositol 3-kinase-AKT-mammalian target of rapamycin; AdoHcy: S-Adenosyl-L-homocysteine; PRMT5: Protein Arginine Methyltransferase 5; VSMCs: Vascular Smooth Muscle Cells; NOX4: NADPH Oxidase 4

Figure 2 summarizes the various ways in which TMAO disrupts metabolism through its powerful signaling mechanisms.

## TMAO and human diseases

Many studies published in the past decade have examined a possible correlation between elevated TMAO and various human diseases. In vitro studies have been able to quantify some of the signaling effects of TMAO and to hypothesize mechanisms by which it might increase risk.

### Gestational and type-2 diabetes

Individuals with type 2 diabetes have notably higher TMAO levels compared to non-diabetics. Sustained elevated TMAO levels leads to an increased risk of both gestational diabetes and type 2 diabetes (Mohammadi et al., 2025). MIN6 cells are a widely used, immortalized mouse pancreatic β-cell line derived from an insulinoma. They mimic normal β-cells by secreting insulin in response to glucose. Short-term exposure of MIN6 cells grown in culture to TMAO decreased oxidative phosphorylation (OXPHOS) and ATP production, promoted glycolysis, inhibited calcium transients following glucose exposure, and decreased insulin secretion in response to glucose. Long-term exposure of pancreatic islets to TMAO promotes β-cell stress, dedifferentiation, and apoptosis (Kong et al., 2024).

### Fibrosis and systemic sclerosis

Systemic sclerosis, or scleroderma, is a chronic autoimmune disease that causes hardening and tightening of the skin and connective tissues due to excess collagen production. It affects both the vasculature and internal organs, including the lungs, heart, kidneys and digestive tract (Gabrielli et al., 2009). Remarkably, TMAO can reprogram mesenchymal progenitors into scar-forming myofibroblasts in the skin, via the putative TMAO receptor protein R-like endoplasmic reticulum kinase (PERK) pathway. The liver enzyme, FMO3, which oxidizes TMA to TMAO, was found to be upregulated in skin fibroblasts in association with systemic sclerosis (Kim et al., 2022). More generally, chronic exposure to elevated TMAO appears to be associated with an enhanced propensity towards fibrosis in association with several other conditions, including chronic kidney disease, heart failure, and metabolic dysfunction-associated steatotic liver disease (Jang et al., 2024).

This tendency towards fibrosis may be a strategy to sequester excess deuterium in proline residues in collagen molecules. The amino acid proline is highly overrepresented in collagen. Proline has been shown experimentally to be able to trap and sequester deuterium bound to its C1 carbon atom, even under highly acidic conditions (Sletten & R Schoenheimer, 1944). Collagen extracted from grey seals was found to have twice the concentration of deuterium in (hydroxy)proline compared to the concentration in seawater (Gharibi et al. 2022; Boros et al. 2024b).

### Non-alcoholic fatty liver disease

Non-alcoholic fatty liver disease (NAFLD), also known as metabolic dysfunction-associated steatotic liver disease (MASLD), is the most common chronic liver disease in the world. It includes steatosis, steatohepatitis, and liver fibrosis (Byrne & G. Targher, 2015). In a study published in 2016, it was found that plasma TMAO levels of patients with NAFLD (0.434 µM) was 4.17 times higher than that of healthy controls (0.104 µM) (Chen et al., 2016). A meta-analysis analyzing seven studies involving 7583 individuals reached a similar result, that NAFLD is statistically significantly correlated with plasma TMAO levels (Theofilis et al., 2022).

In a laboratory-based study by Yang et al. (2024), adult male zebrafish were fed a diet containing 1–3% TMAO for 20 weeks. The researchers observed that TMAO caused lipid accumulation, inflammatory infiltration, liver injury and fibrosis in the livers of the zebrafish. They also confirmed that the PERK signaling pathway was upregulated in response to TMAO. After binding with PERK in the liver, TMAO activates the PERK branch of the unfolded protein response, inducing the transcription factor FoxO1, a key driver of metabolic disease (Chen et al., 2019).

### Cardiovascular disease and heart failure

TMAO is strongly linked to atherosclerosis, through its promotion of vascular inflammation, foam cell formation, suppression of reverse cholesterol transport, and facilitation of cholesterol accumulation in the artery wall, increasing risks for heart attack, heart failure, and cardiovascular death (Salzano et al., 2020; Wang et al., 2021; Zhang et al., 2021; Crisci et al., 2023). Strong evidence shows that TMAO is linked to poor outcomes, increased mortality, and worse prognosis in heart failure patients, even independent of kidney function, with higher levels indicating greater risk for major adverse cardiac events (Li et al. 2020, 2022b; Crisci et al. 2023; Jarmukhanov et al. 2024).

Just since 2024, multiple review studies have focused on the gut-heart axis, linking gut dysbiosis to heart failure and pointing to TMAO as a marker and potentially a therapeutic target (Jarmukhanov et al., 2024; Shariff et al., 2024; Albulushi et al., 2025; Makieh et al., 2025; Abdulrahim et al., 2025). Gut dysbiosis causes a leaky gut barrier which leads to the release of bacterial metabolites and endotoxins, such as LPS, that promote systemic inflammation (Di Vincenzo et al., 2024). While elevated levels signal higher cardiovascular risk, it is still unclear whether TMAO is a direct cause or just a marker (Oktaviono et al., 2023). Elevated plasma levels of TMAO are correlated with accelerated atherosclerosis, increased platelet reactivity, and heightened potential for thrombosis (Zhu et al., 2016).

### Preeclampsia

Preeclampsia (PE) is a relatively common condition during pregnancy which is characterized by hypertension developing late in pregnancy, along with evidence of maternal organ failure, fetal growth restriction, and even stillbirth if not properly treated. Multiple studies confirm that PE is strongly associated with significantly increased maternal serum levels of TMAO. Elevated TMAO levels are not just a marker but an active participant in the progression of PE (Mubeen et al., 2025).

Gut dysbiosis associated with increased plasma levels of lipopolysaccharide and TMAO were found in patients with PE (Wang et al., 2019). Patients with PE often having fewer SCFA-producing bacteria and low Akkermansia colonization, leading to reduced production of butyrate associated with increased inflammation (Zong et al., 2023). A study on human placental explants demonstrated that TMAO induced increased expression of NOX in those cells, along with increased production of ROS (Chang et al., 2021).

### Dementia

Neuroinflammation is a characteristic feature of Alzheimer’s disease (AD) as well as many other neurodegenerative diseases (Guzman-Martinez et al., 2019). We have seen that TMAO induces an inflammatory response by activating the NLRP3 inflammasome and NF-κB, releasing inflammatory cytokines and promoting oxidative stress in association with atherosclerosis and IBD (Oktaviono et al., 2023; Wang et al., 2025). TMAO is elevated in the cerebrospinal fluid of patients suffering from AD (Arrona Cardoza et al., 2022).

In an experiment conducted on 410 individuals binned into three groups of diagnosed AD, mild cognitive impairment (MCI), and unimpaired, levels of TMAO in cerebrospinal fluid were compared among the three population groups. The *p*-value for the comparison of the control group vs. MCI was 0.02, and for the control group vs. AD was 4.1E-6. After controlling for age and sex, levels of TMAO in the cerebrospinal fluid were significantly positively correlated with several markers for AD, including phosphorylated tau (p-tau), the ratio of p-tau to Aβ42, total tau, and neurofilament light chain protein (Vogt et al., 2018).

In a study by Hu et al. (2023), a group of mice were induced with dementia by exposure to D-galactose and aluminum chloride. A subset was then exposed to TMAO, and the mice were examined for changes in learning, memory, histopathology, inflammatory factors, and PI3K/Akt/mTOR signaling. They found that TMAO increased the release of inflammatory cytokines and promoted the PI3K/Akt/mTOR signaling pathway.

Mice treated with TMAO (1.5% concentration in water) for 16 weeks developed neuronal senescence in the hippocampal CA3 region, associated with oxidative stress and cognitive impairment. The effects on mitochondria in the hippocampal CA1 region were profound, including swelling and deformation of the mitochondria, a reduction in the number of cristae, and an accumulation of lipofuscin within the cells (Li et al., 2018).

### Cancer

Elevated plasma TMAO is strongly associated with colon cancer, and is also linked to other types of cancer, including prostate cancer, oral cancer, breast cancer, pancreatic cancer, and liver cancer. TMAO promotes inflammation, oxidative stress, cell proliferation and angiogenesis, all hallmarks of cancer (Saha et al., 2025). Exposure of colorectal cancer cells grown in vitro to TMAO led to enhanced secretion of vascular endothelial growth factor A (VEGFA) with increased tumor proliferation (Yang et al., 2022).

In a review article reporting on six observational studies that examined associations of TMAO with cancer risk, the following ORs were provided: colorectal cancer: 1.49, prostate cancer: 1.36, primary liver cancer: 2.85, and pancreatic cancer: 2.36 (Li et al. 2022a). A strong association between breast cancer and plasma TMAO was found in a metabolomics study (Morad et al., 2022). Prostate cancer was the fourth most diagnosed cancer worldwide in 2022. In vitro, it was shown that TMAO enhances the proliferation and migration of prostate cancer cells by upregulating heme oxygenase 1 via activation of the p38 MAPK signaling pathway (Zhou et al., 2025).

PC-3 cells are a widely used human prostate cancer cell line, which is used to seed prostate cancer in mice. In an experiment where mice were injected with PC-3 cells to induce prostate cancer, a comparison was made on the rate of tumor growth and metastasis in mice fed a low choline vs. a high (synthetic) choline diet. The high-choline diet group had significantly more lung metastases and a larger tumor mass in the prostate gland (Zhou et al., 2025).

Several papers have reported beneficial results in treating various cancers with deuterium depleted water (DDW) (Boros et al., 2021; Lu & Chen, 2024). Two papers published in 2025 presented arguments that cancer cells are able to concentrate deuterium internally and release into the external environment both deupleted protons and deupleted nutrients, such as lactate. Cancer cells actually provide the resident immune cells with beneficial low-deuterium nutrients that can support mitochondrial health (Seneff and Kyriakopoulos 2025a; Kyriakopoulos and Seneff 2026). Even when their mitochondria are healthy, cancer cells derive most of their ATP through glycolysis via the Warburg effect (Liberti & Locasale, 2016), thus minimizing the need for the ATPase nanomotors that are vulnerable to deuterium toxicity.

**Fig. 3:**
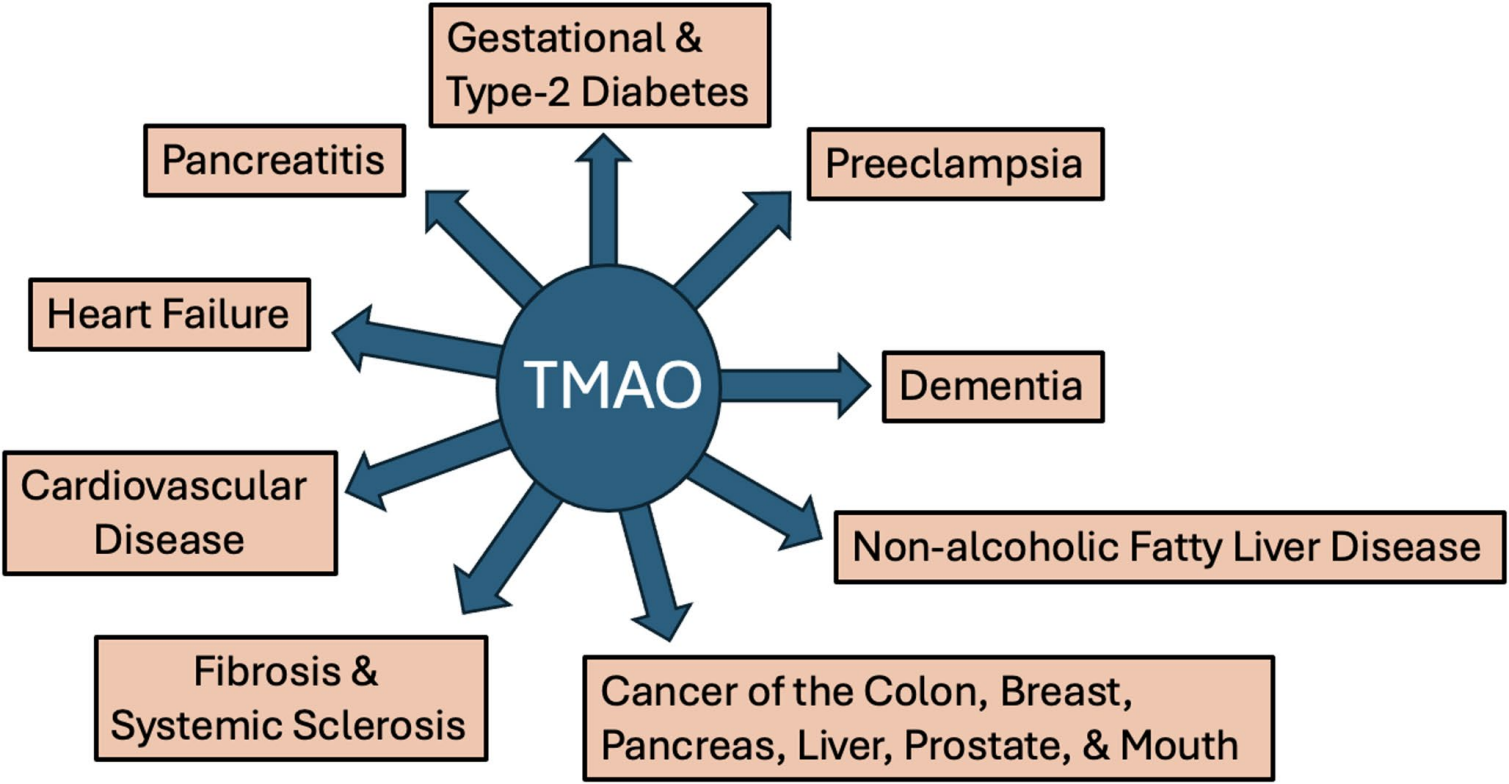
Some of the diseases that have been associated with elevated levels of plasma TMAO. TMAO: Trimethylamine N-Oxide

Figure 3 illustrates several diseases that have been linked to elevated plasma levels of TMAO. 

## Does oxidative stress lead to mitochondrial deupletion?

While oxidative stress is a major contributor to cellular damage, the processes involved in resolving ROS are an essential part of the mechanism by which the cell reduces deuterium levels in the mitochondria. Intracellular ROS are derived mainly from NOX, xanthine oxidase, and the mitochondrial electron-transport chain (mETC). Excess mitochondrial deuterium promotes increased ROS generated by the mETC (Olgun, 2007). Superoxide dismutase (SOD) converts ROS to H_2_O_2_, which can release the highly destructive hydroxyl radical in the presence of reduced iron (Fe2+) (Endale et al., 2023; Sies et al., 2017). However, H_2_O_2_ is an excellent source of DDW in the mitochondria, as long as there is sufficient mitochondrial glutathione and both glutathione peroxidase and glutathione reductase are adequately expressed. H_2_O_2_ freely crosses the mitochondrial membrane, and, with adequate antioxidant support, it is rapidly converted to two molecules of DDW, catalyzed by glutathione peroxidase (Seneff and Kyriakopoulos 2025b).

An in vitro experiment published in 2020, where differentiated PC12 cells (a popular model for neurons) were exposed to H_2_O_2_, directly demonstrated the protective effects of DDW against oxidative stress. These authors wrote: “The results indicated that DDW could attenuate H_2_O_2_-induced apoptosis, reduce ROS formation, and increase CAT, CuZn-SOD and SOD activity in H_2_O_2_-treated PC12 cells” (Wu et al., 2020).

## Do lipid-laden foam cells support mitochondrial deupletion?

We have seen that one of the most striking effects of plasma TMAO is the ability of TMAO signaling to promote the accumulation of lipids in atherosclerotic plaque. Ox-LDL, produced through ROS attack on LDL particles, plays a key role in atherosclerosis. Ox-LDL is taken up by immune cells adhering to the artery wall via scavenger receptors, and it directly leads to their conversion into lipid-laden foam cells (Mosalmanzadeh & Pence, 2024).

The induction of an inflammatory response by TMAO, especially in the presence of reactive metals, results in a lipid peroxidation chain reaction that produces a large number of derived lipid products with powerful signaling capabilities (Oktaviono et al., 2023). A large class of eicosanoids are produced through the upregulation of lipoxygenase (LOX) and cycloxygenase (COX), including leukotrienes, prostaglandins, prostacyclins, and thromboxanes, all of which are powerful signaling molecules that can launch an inflammatory cascade (Lone & K Taskén, 2013). The oxidative metabolism of arachidonic acid (AA) that is released from membrane phospholipids through the catalytic activity of phospholipase A2 (PLA2) generates various eicosanoids that induce further ROS generation by stimulating NOX in a positive feedback loop (Cho et al., 2011).

Polyunsaturated fatty acids (PUFAs) are a major component of lipid droplets. They are highly sensitive to ROS, which leads to oxidative attack, specifically on their vulnerable bis-allylic carbon atoms. Bis-allylic carbon atoms are best defined through the molecular formula “…C = C-C*-C = C…”; i.e., they are a carbon atom bonded to two other carbon atoms in a chain, where both neighbors are double-bonded to the other adjacent carbon atom. Lipid peroxyl radicals (LOO$\cdot$) participate in a chain reaction to produce new lipid free radicals in a “free-for-all” reaction cascade that eventually resolves with the synthesis of anti-inflammatory molecules called resolvins and lipoxins (Janakiram & Rao, 2009; Kahnt et al., 2023; Babakr, 2025).

Bis-allylic carbon atoms are far more labile than other carbon atoms in fatty acids, which means that they may freely exchange their protons with deuterons from the aqueous medium, especially under conditions of oxidative stress. Arachidonic acid (AA) is a common PUFA containing three bis-allylic carbon atoms, at C7, C10, and C13. Interestingly, it has been discovered that deuteration of the C10 bis-allylic carbon atom in AA causes a significant increase in the production of protective lipoxin A4/B4 (LXA4/LXB4) by macrophages, likely because deuteration suppresses COX activity due to a large deuterium KIE, redirecting the reaction towards LOX activity instead (Navratil et al., 2018). COX enzymes are unable to abstract the C10 deuteride, and this favors the LOX pathway, ultimately arresting the reaction cascade.

Since lipid peroxidation ultimately leads to further production of H_2_O_2_, which can then be converted to DDW in the mitochondria, it is tempting to speculate that atherosclerotic plaque serves a useful purpose in supplying mitochondria in the heart with DDW, to support mitochondrial health. We hypothesize that, as the chain reaction draws to a close, many of the lipids in the foam cells have acquired deuterium at their bis-allylic carbon atoms, which serves both to quench the chain reaction and to sequester deuterium atoms to keep them from reaching the mitochondria (Seneff et al. 2025b).

It has been shown experimentally that PUFAs that are deuterated at their bis-allylic carbon atoms offer therapeutic value in protection from mitochondrial oxidative stress, and supplementing with deuterated PUFAs is even being considered as a therapeutic option (Andreyev et al., 2015).

## Archaeobiotics

Methanogenic archaea typically constitute about 10% of the total archaeal community in the gut (Samuel et al., 2007). It was first observed in the 1980 s that adults with IBD rarely excrete methane gas (McKay et al., 1985). Methanogens are strictly anaerobic archaea and are traditionally considered highly sensitive to oxidative stress. When exposed to ROS such as hydrogen peroxide or superoxide, their growth and methane-producing activities are significantly suppressed (Cisek et al., 2024).

Much can be learned from experiments involving anaerobic digestion of sewage sludge. In a study specifically addressing the problem of methanogenesis interfering with the production of volatile fatty acids during anaerobic digestion, the authors found that methanogens are highly sensitive to H_2_O_2_. These authors wrote: “The light-exposed H_2_O_2_ elevated intracellular ROS levels, leading to a sharp decline in methanogen abundance (only 5.02% remained compared to the inoculum). Firmicutes became dominant, increasing from 14.03% (inoculum) to 52.35% under high oxidative stress” (Sun & He, 2025). Oxidative stress is a central feature of IBD, stemming from an imbalance where excessive production of ROS overwhelms antioxidant defenses (Muro et al., 2024). In both ulcerative colitis and Crohn’s disease, an increased fecal abundance of Firmicutes at the phylum level was observed, compared to controls (Alam et al., 2020).

Methanomassiliicoccales is a distinct order of Archaea, only discovered in 2012, which were then added as a 7th order of methanogens (Brugère et al., 2014). Jean-François Brugère et al. (2014) proposed that therapeutic use of these archaea might be a good strategy for reducing plasma levels of TMAO and thus treating cardiovascular disease. These authors confirmed that Methanomassilicoccales strains inhabiting the human gut indeed metabolize TMA to produce methane gas. By analyzing the genomics of several species from the 7th order, they determined that many of these species harbor the genes coding for the enzymes that metabolize TMA to produce methane gas. These authors wrote: “Collectively this suggests to us that natural methanogenic inhabitants of the human gut will be able to metabolize TMA, and could deplete this metabolite as it is formed by bacterial elements of the microbiota.”

Methylotrophs metabolize methylated amines, using hydrogen gas to reduce the methyl groups to methane, producing ammonia as a by-product. Methylotrophs compete with methanogens for the hydrogen gas, but they only require one molecule of hydrogen, as opposed to four required by methanogens, whose substrate is CO_2_. However, methylotrophs consume hydrogen down to partial pressures < 0.1 Pa, whereas the threshold values for methanogens typically range from 2.8 to 10 Pa. Thus, the threshold for methylotrophs is up to two orders of magnitude lower than the threshold for methanogens. Methylotrophs significantly outcompete methanogens for hydrogen, and their activity is only limited by the availability of methyl groups (Feldewert et al., 2020).

## A crucial role for *A. muciniphila*

We have already shown that *A. muciniphila* are an important symbiont in the gut which are beneficial for gut health and which release a protein that stimulates the secretion of GLP-1 by the L-cells lining the colon, curbing appetite and offering protection from diabetes. *Faecalibacterium prausnitzii* (*F. prausnitzii*) is a crucial, abundant, anti-inflammatory bacterium in the human gut, known for producing butyrate and promoting intestinal health, but it is often depleted in IBD, acting as a key indicator of gut dysbiosis (Parsaei et al., 2021). *A. muciniphila* degrades host-derived sulfomucins into small organic molecules that *F. prausnitzii* then utilizes to produce butyrate (Belzer et al., 2017).

Cross-feeding between *A. muciniphila* and anaerobic microbes is essential for maintaining gut health. By breaking down the sulfomucins produced by goblet cells, *A. muciniphila* provide small organic molecules that the strict anaerobes can use to produce H_2_ and CO_2_. Methylogenic archaea then use a hydrogen-dependent methylotrophic pathway to produce methane gas from TMA, thereby further fractionating out the deuterium and preventing the TMA from reaching the liver and the circulation as TMAO.

In a paper published in 2016, APOE-/- mice on normal chow diet or on a Western diet were treated with *A. muciniphila* by daily oral gavage for 8 weeks, and this was followed by histological evaluations of atherosclerotic lesions in the aorta. They found that *A. muciniphila* prevented Western diet-induced inflammation in both the circulation and local atherosclerotic lesions, as evidenced by reduced macrophage infiltration and decreased expression of proinflammatory cytokines and chemokines. These changes were accompanied by a marked attenuation in metabolic endotoxemia (Li et al., 2016).

**Fig. 4:**
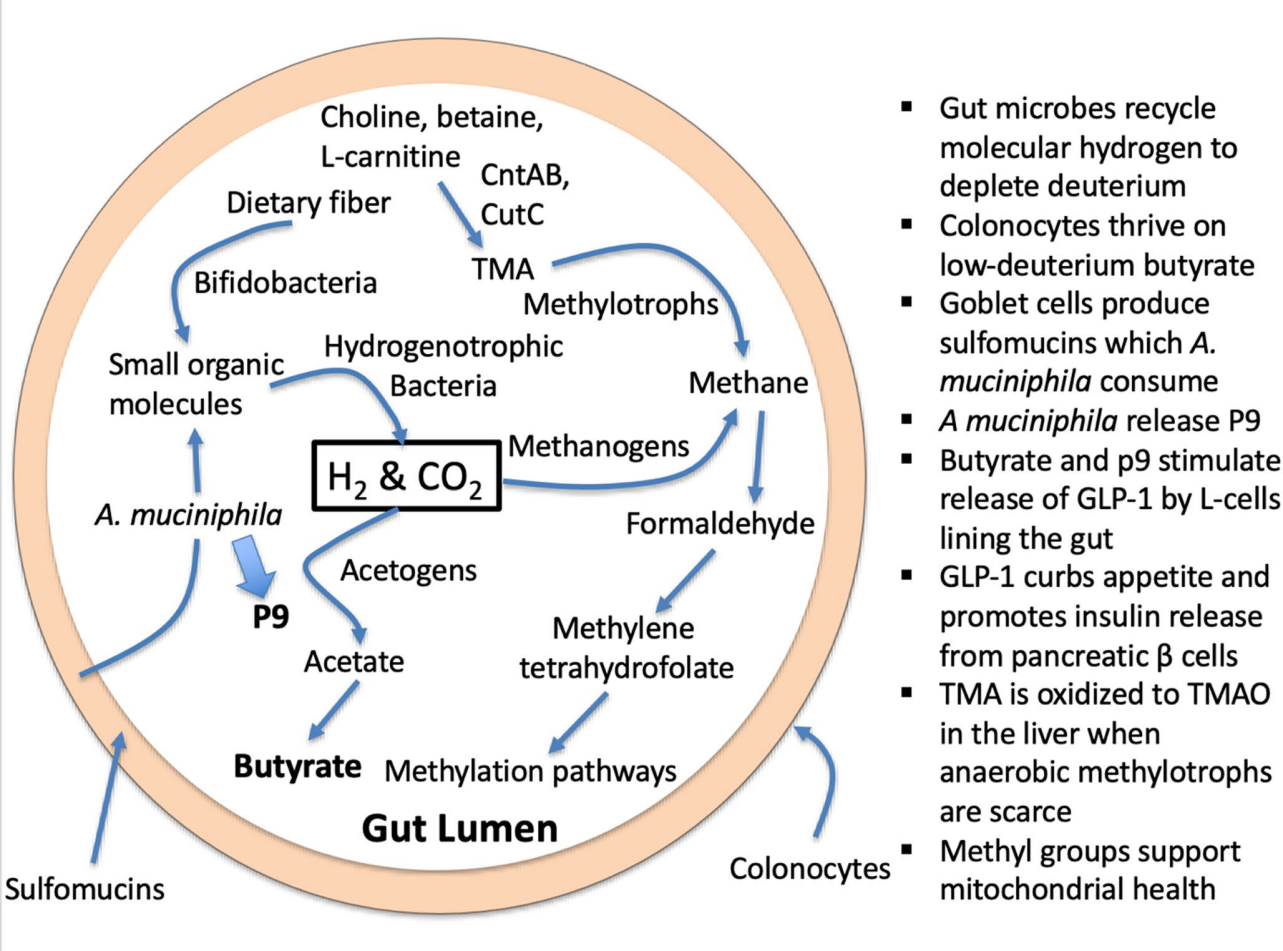
A schematic diagram of microbial metabolic pathways involved in assuring an adequate supply of deupleted nutrients to the host. TMA: Trimethylamine; CntAB: Carnitine monooxygenase; CutC: Choline TMA-lyase. GLP-1: Glucagon-Like Peptide-1. *A. muciniphila: Akkermansia muciniphila*

*A. muciniphila*-mediated reduction in circulating endotoxin levels could be attributed to the induction of intestinal expression of the tight junction proteins zona occludens protein-1 and occludin. The protection against chronic inflammation strengthened the gut barrier and protected against cardiovascular disease. Long-term infusion of endotoxin to APOE-/- mice reversed the protective effect of *A. muciniphila* against atherosclerosis (Li et al., 2016). In other studies, colonization by *A. muciniphila* has been found to be inversely related to the plasma levels of TMAO (Luo et al., 2022). This is likely because the anaerobic archaea that fully metabolize TMA are highly sensitive to inflammation.

Figure 4 schematizes the various pathways by which the gut microbes work in symbiosis with the host cells to maintain a supply of deupleted nutrients (primarily acetate, butyrate, and methyl groups) for the host mitochondria.

## Strategies to lower TMAO levels

It is clear that elevated plasma TMAO is a risk factor for a broad range of chronic diseases, and therefore it is compelling that a strategy that reduces plasma TMAO should show health benefits. However, simply avoiding foods that provide precursors to TMAO is not likely to be productive. Choline, L-carnitine, and betaine are the primary sources that fuel the methylation pathway. Eggs and seafood, rich sources of these nutrients, also contain many valuable micronutrients and healthy fats that are also essential.

The pharmaceutical industry is currently developing therapeutic drugs that suppress the microbial enzymes, e.g., choline-TMA lyase (CutC) and carnitine monooxygenase (CntAB), that produce TMA from its natural precursors (Witkowski et al., 2020; Li et al., 2021). This is also not likely to be successful, because the microbial pathway that derives methane gas, and ultimately methyl groups, from TMA precursors is an essential component of the methyl-group recycling process that helps assure a good supply of deupleted methyl groups to the host. Choline is recognized as an essential nutrient for pregnant women, who often consume a diet that is deficient in choline. A rich supply of choline in prenatal life facilitates normal brain development and improves neural and cognitive function (Korsmo et al., 2019; Derbyshire et al., 2020).

An important factor to consider is the difference between natural and synthetic TMA precursors. Choline bitartrate is a popular supplement based on synthetic choline, but it is likely defective because the methyl groups are not deupleted. It is possible to take natural phosphatidylcholine as a supplement, and this is probably beneficial. Lecithin derived from soy, sunflower or egg yolk is naturally rich in phosphatidyl choline, and it has been shown to have significant health benefits (Onaolapo et al., 2024). However, it is likely preferable to assure an adequate supply of dietary choline through food choices.

Several herbal supplements have been shown to be effective in reducing TMAO levels. Berberine, baicalin, and curcumin are potent plant compounds known for strong anti-inflammatory and antioxidant effects, as well as metabolic support. These supplements were shown to lower TMAO levels by modifying the composition of the gut microbiome (Qin et al., 2025). In both human and mouse studies, allicin (a potent sulfur compound in garlic) lowered plasma TMAO levels, improved gut microbial diversity, and increased the relative abundance of beneficial bacteria (Panyod et al., 2022).

Deuterium depleted water (DDW) is commercially available, at dilution levels as low as 5 ppm. It can be mixed with tap water to simulate natural glacier water, typically containing around 100 ppm deuterium. Although the number of studies on the effects of therapeutic deuterium depletion on various health conditions is small, a review paper found that deuterium depletion has shown promise in preventing and treating cancer, improving long-term memory, enhancing sports performance, and reducing symptoms of depression (Korchinsky et al., 2024).

It is apparent that the best way to reduce TMAO levels, while simultaneously boosting methylation supplies, is to promote an abundant colonization of anaerobic archaea in the gut, so that they can clear (fully metabolize) the TMA before it has a chance to become TMAO. Pesticides, including insecticides, herbicides, fungicides, and toxic metals, are known to negatively impact the gut microbiome (Ali & AlHussaini, 2024). Consuming a certified organic whole foods diet that is rich in prebiotics (e.g., fiber), probiotics, sulfur-containing foods (as precursors to glutathione and sulfomucins), and foods that are rich in animal-based fats (low-deuterium nutrients) and micronutrients (vitamins, minerals and antioxidants) is the best approach to sustain good health and longevity.

## Discussion

TMAO is a fascinating small molecule with powerful signaling effects, whose plasma elevation is associated with many chronic diseases, most notably diabetes, fatty liver disease, and cardiovascular disease, but also preeclampsia, dementia, and many types of cancer (Liu et al., 2025). Certain members of the gut microbiome synthesize its predecessor, TMA, from dietary sources, including L-carnitine, choline, and betaine, and the liver enzyme FOM3 oxidizes TMA to TMAO and releases it into the circulation (Tacconi et al., 2023). In this paper, we develop the argument that TMAO serves as a marker for excess deuterium in the methylation pathway, and, by extension, in the mitochondria, systemically. We hypothesize that the alterations in metabolism it induces, although they produce damaging ROS, often serve as a source of deupleted protons to help restore cellular mitochondrial health.

The 2011 paper that first proposed a significant role for TMAO in disease was based on feeding mice synthetic phosphatidylcholine containing three fully deuterated methyl groups. Inadvertently, these authors demonstrated the powerful effect of deuterium loading in the methylation pathway (Wang et al., 2011). A later paper used deuterated L-carnitine to demonstrate a similar sharp elevation in plasma TMAO (Koeth et al., 2013). By contrast, methyl groups produced naturally by the gut microbes are likely severely deupleted (Seneff et al. 2025c). And human consumption of several eggs every day does not raise plasma TMAO levels, despite their rich supply of natural choline and L-carnitine (Wilcox et al., 2021). While methyl groups have powerful epigenetic effects, the ultimate fate of methyl groups is their metabolism to CO_2_ and water that is most likely deuterium depleted in the mitochondria (Rosenberger et al., 2021).

We hypothesize that a crucial role for the gut microbiome is the constant recycling of severely deupleted molecular hydrogen, as a means to strip deuterium from basic nutrients. The microbial enzyme that converts the methyl groups in TMA to formaldehyde has a very high deuterium KIE, leaving behind deuterium rich TMA which later gets oxidized to TMAO (Wanninayake et al., 2019). When the molecular hydrogen recycling system is disrupted, the colonocyte mitochondria become impaired, likely due to deuterium overload.

A microbial imbalance leading to reduced colonization by beneficial bacteria and an overgrowth of pathogenic species is the primary cause of overproduction of TMAO. IBD is associated with ROS that interfere with the survival of anaerobic archaea, the species that are critical for metabolizing TMA (Cisek et al., 2024). While inflammation is linked to many chronic diseases, what is not fully appreciated is the fact that the lipid peroxidation chain reaction is critical both for providing DDW and for sequestering deuterium at the bis-allylic carbon atoms in PUFAs such as arachidonic acid. The inflammatory response is ultimately resolved with the production of resolvins and lipoxins, once the lipid deposits are sufficiently deuterated (Seneff et al. 2025b, c). When there are abundant levels of SOD, catalase, glutathione, glutathione peroxidase and glutathione reductase, the production of superoxide becomes a powerful resource for restoring mitochondrial health by supplying the mitochondria with DDW. Even in the healthy state, the peroxisome produces deupleted H_2_O_2_ that is delivered to the mitochondria, presumably as a source of DDW (Seneff and Kyriakopoulos 2025b).

Table 2 provides a list of key concepts which support our hypotheses that methyl groups supply deupleted protons to the ATPase nanomotors and that TMAO is a causal factor in many chronic diseases that are linked to mitochondrial dysfunction.

**Table 2:** Biological facts and associated references supporting the hypothesis that methylation pathways carry deuterium depleted protons and that TMAO (hypothesized to act as a signal for an imbalanced microbiome and deuterium overload in mitochondria) is a causal factor for many diseases and conditions. TMAO: trimethylamine oxide; TMADH: trimethylamine dehydrogenase; KIE: kinetic isotope effect

Biological fact	Reference
Deuterium is damaging to the mitochondrial ATPase nanomotors	Olgun, 2007
Heavy water inhibits DNA double-strand break repairs	Yasuda et al., 2024
Mitochondrial DNA double-strand break repair proteins have high deuterium KIEs	Werner & Stivers, 2000
A microbial hydrogenase produces molecular hydrogen that has lost 80% of the deuterium	Krichevsky et al., 1961
Hydrogenase enzymes expressed by gut microbes produce molecular hydrogen from small organic molecules	Vignais, 2008
Microbial hydrogenases can have a deuterium KIE as high as 43 in an acidic environment	Greene et al., 2015
Methylene-tetrahydrofolate (CH2-THF) is produced from formaldehyde, which is derived from methane gas, which is derived from molecular hydrogen and carbon dioxide	Pietzke et al., 2020
Methyl-tetrahydrofolate donates its methyl group to homocysteine to synthesize methionine, the universal methyl donor	Matthews et al., 1998
Methylation is a universal biochemical process which covalently adds methyl groups to a variety of molecular targets	Menezo et al., 2020
Phosphatidyl-choline carries a trimethylamine unit whose methyl groups all come from S-adenosylmethioninine (SAMe)	Li et al., 2023
Dietary choline, betaine, and L-carnitine are metabolized by gut microbes to produce trimethylamine (TMA)	Tang & Hazen, 2014
The anaerobic archaeal enzyme that metabolizes TMA (TMADH) is a flavoprotein with a high deuterium KIE (around 8.6)	Basran et al., 2001, Wanninayake et al., 2019
Supplementing humans with D9-PC (fully deuterated methyl groups) sharply increased plasma and urine levels of D9-TMAO	Tang et al., 2013
Deuterated L-carnitine supplementation in rats raises serum TMAO levels	Koeth et al., 2013
Mitochondrial health critically depends on 1C metabolism	Rosenberger et al., 2021
Deuterated bis-allylic carbon atoms arrest the lipid peroxidation chain reaction	Seneff et al. 2025b, c
Fatty acids are depleted in deuterium	Evans & Beharie, 2024
TMAO is a risk factor for cardiovascular disease	Amaritei et al., 2025
TMAO is a risk factor for heart failure and colorectal cancer	Subramaniam & Fletcher, 2018
TMAO is a risk factor for Alzheimer’s disease	Arrona Cardoza et al., 2022
TMAO is a risk factor for many different types of cancer	Saha et al., 2025
TMAO is a causal factor, and not just a marker, for disease	Liu et al., 2025
TMAO induces a lipid peroxidation chain reaction	Oktaviono et al., 2023

## Conclusions

In this paper, we have shown that TMAO, a causal factor for many diseases, may act as a marker for gut dysbiosis and for excess deuterium load in mitochondria, systemically. We traced through many of the biological pathways involving 1C metabolism and showed the integral role that gut bacteria play in stripping deuterium from the methyl groups. We conclude that the best way to maintain good health and longevity is through consumption of healthy foods, which includes mainly animal-based fats that are naturally deupleted, sulfur-containing foods, and nutrient dense foods such as eggs and seafood that are rich in choline and L-carnitine. With moderation, prebiotics (e.g., fiber), probiotics, and antioxidants may also be considered. It is important to consume certified organic foods to reduce exposure to toxic pesticides which can disrupt the gut microbiome. The Western diet, primarily based on industrially produced heavily processed foods, contaminated with pesticides, is likely a major cause of the increase we have seen in recent decades in the prevalence of chronic disease.

## Data Availability

No datasets were generated or analysed during the current study.

## Declarations

### Artificial intelligence assistance

During the preparation of this manuscript, the authors used Google’s Gemini 3, solely for structuring and organization of ideas and literature search assistance.
